# Enhancement of Antibacterial Activity of Capped Silver Nanoparticles in Combination with Antibiotics, on Model Gram-Negative and Gram-Positive Bacteria

**DOI:** 10.1155/2013/871097

**Published:** 2013-07-18

**Authors:** Aruna Jyothi Kora, Lori Rastogi

**Affiliations:** National Centre for Compositional Characterisation of Materials (NCCCM), Bhabha Atomic Research Centre, Hyderabad 500 062, India

## Abstract

The nanoparticles used in this study were prepared from AgNO_3_ using NaBH_4_ in the presence of capping agents such as citrate, sodium dodecyl sulfate, and polyvinylpyrrolidone. The formed nanoparticles were characterized with UV-Vis, TEM, and XRD. The generation of silver nanoparticles was confirmed from the appearance of yellow colour and an absorption maximum between 399 and 404 nm. The produced nanoparticles were found to be spherical in shape and polydisperse. For citrate, SDS, and PVP capped nanoparticles, the average particle sizes were 38.3 ± 13.5, 19.3 ± 6.0, and 16.0 ± 4.8 nm, respectively. The crystallinity of the nanoparticles in FCC structure is confirmed from the SAED and XRD patterns. Also, the combined antibacterial activity of these differently capped nanoparticles with selected antibiotics (streptomycin, ampicillin, and tetracycline) was evaluated on model Gram-negative and Gram-positive bacteria, employing disc diffusion assay. The activity of the tested antibiotics was enhanced in combination with all the stabilized nanoparticles, against both the Gram classes of bacteria. The combined effects of silver nanoparticles and antibiotics were more prominent with PVP capped nanoparticles as compared to citrate and SDS capped ones. The results of this study demonstrate potential therapeutic applications of silver nanoparticles in combination with antibiotics.

## 1. Introduction

Since ancient times, silver has been known to possess antibacterial properties [1], but the solubility characteristics of silver metal and silver salts (e.g., silver nitrate) render it impractical in many clinical scenarios, that is, where silver nanoparticles (Ag NPs) have been a subject of great interest among researchers [2–4], because it is not only facile to synthesize silver nanoparticles of desired sizes [5, 6] and shapes [7–9] dispersed in aqueous/organic phases but also feasible to make films, with the composite of these particles suiting various applications in the field of medical diagnosis and therapy. The use of silver nanoparticles in materials modification for application in different fields such as clothing, semiconductor, and preparation of nanocomposite materials with improved performances has been demonstrated. For example, silver nanoparticles have been successfully coated on medical devices for infection-free transplantation [10, 11]. Silver nanoparticles have also been coated on various fabrics [12–15]; the coating of nanosilver imparts not only the metallic feature to the fibers rendering the textiles conductive but also the antibacterial property to the textiles. These studies suggest that it is possible to have extended action of silver-nanoparticle-based antibacterial activities. Moreover, it can be expected that the high specific surface area and high fraction of surface atoms of silver nanoparticles will lead to high antimicrobial activity as compared with bulk silver metal.

In recent years, resistance to antibiotics by pathogenic bacteria and fungi has been increasing at an alarming rate and has become a serious problem [16, 17]. For example, *S. typhi* has exhibited resistance to antibiotics like chloramphenicol, ampicillin, quinolone, and trimethoprim. And also *E. coli* shows resistance to a variety of antibiotics like ampicillin, kanamycin, sulfisoxazole, streptomycin, tetracycline, ticarcillin, and so forth. Silver has been thought of as a promising agent for overcoming the resistance mechanism of antibacterial action on a range of targets as compared to a specific site of action in the case of antibiotics [18–20]. Hence, nanoparticle-based antibacterial formulations could be effective bactericidal materials as they will exhibit combined effects of silver and antibacterial agents. The enhanced activity of silver nanoparticles and antibiotics together has been reported earlier [21–25]. Herein, in this study we have compared the effect of three different capping agents citrate, sodium dodecyl sulfate, and polyvinyl pyrrolidone on the synthesis of silver nanoparticles. The nanoparticles used in this study were prepared by a common synthetic route that is borohydride reduction of silver nitrate in the presence of stabilizing agents. One of the important criterions for nanoparticle production is the prevention of particle aggregation during synthesis. The nanoparticles can be stabilized either by steric or electrostatic forces. The steric stabilization can be achieved by adsorbing polymers such as polyvinyl pyrrolidone, whereas the electrostatic stabilization can be attained by surface modifiers such as sodium dodecyl sulfate and citrate. The studies were further extended to investigate the combined antibacterial effect of these differently capped silver nanoparticles with antibiotics on both the Gram classes of bacteria.

## 2. Materials and Methods

### 2.1. Synthesis of Silver Nanoparticles

Silver nitrate (AgNO_3_), sodium borohydride (NaBH_4_) (E. Merck, Mumbai, India), trisodium citrate dihydrate (Finar, Hyderabad, India), sodium dodecyl sulfate (SDS) (SRL, Mumbai, India), and polyvinylpyrrolidone (PVP) with average M Wt 40,000 (Sigma-Aldrich, Mumbai, India) of analytical reagent grade were used for the synthesis. All the solutions were prepared in ultrapure water. The silver nanoparticles of 1.0 mM concentration were prepared using 6.0 mM of ice cold NaBH_4_, in the presence of 0.4 mM sodium citrate, 0.4 mM SDS, and 0.1% PVP [26], by stirring for 30 min. The produced colloidal solutions were diluted eight times with ultrapure water and the spectra were recorded. 

### 2.2. Characterization of Synthesized Silver Nanoparticles

The UV-visible absorption spectra of the prepared colloidal solutions were recorded using an Elico SL 196 spectrophotometer (Hyderabad, India), from 250 to 800 nm, against blank. The size and shape of the nanoparticles were obtained with JEOL 2100 (Tokyo, Japan) transmission electron microscope, operating at 200 kV. The samples were prepared by depositing a drop of colloidal solution on a carbon coated copper grid and drying at room temperature. The X-ray diffraction analysis was conducted with a Rigaku, Ultima IV diffractometer (Tokyo, Japan) using monochromatic Cu K*α* radiation (*λ* = 1.5406 Å) running at 40 kV and 30 mA. The intensity data for the nanoparticle solution deposited on a glass slide was collected over a 2*θ* range of 35–85° with a scan rate of 1°/min. The hydrodynamic diameter and zeta potential values of the produced silver nanoparticles were assessed with a Malvern Zetasizer Nanosystem (Worcestershire, UK).

### 2.3. Disc Diffusion Assay

All the glassware and media used were sterilized in an autoclave at 121°C for 20 min. The bacterial strains *Escherichia coli* (ATCC 25922) and *Staphylococcus aureus* (ATCC 25923) were used as model test strains for Gram-negative and Gram-positive bacteria, respectively. The bacterial suspension was prepared by growing a single colony overnight in nutrient broth and by adjusting the turbidity to 0.5 McFarland standard. The disc diffusion method was used to evaluate the antibacterial activity of silver nanoparticles in combination with antibiotics. Based on the CLSI standard, the selected concentrations of antibiotics were streptomycin (10 *μ*g), ampicillin (10 *μ*g), and tetracycline (30 *μ*g), respectively [27]. By using spread plate method, the Mueller-Hinton agar plates were inoculated with the turbidity adjusted bacterial suspension, and antibiotic impregnated sterile discs of 6 mm diameter (HiMedia Chemicals Pvt. Ltd., Mumbai, India) were placed on the medium surface. Also, the antibiotic discs loaded with 5 *μ*g of nanosilver were placed on the inoculated plates to see the combined activity. The plates were maintained with discs containing silver nanoparticles and capping agents separately. These plates were incubated at 37°C for 24 h, and zone of inhibition (ZOI) was measured by subtracting the disc diameter from the total inhibition zone diameter. This assay was performed in triplicate.

## 3. Results and Discussion

### 3.1. UV-Visible Spectroscopy (UV-Vis)

**Figure 1:**
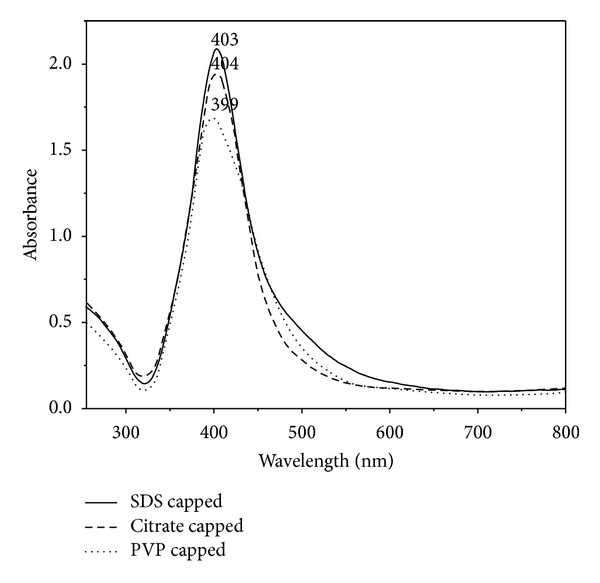
The UV-Vis absorption spectra of capped silver nanoparticles synthesized with sodium borohydride.

The appearance of yellow colour in the reaction mixtures was observed within minutes, an obvious indication for the silver nanoparticle formation. Furthermore, the nanoparticle synthesis was assured by monitoring the absorption spectra of synthesized colloidal solutions, against respective capping agent blanks (Figure 1). In the UV-Vis spectra, a single strong peak was observed for citrate, SDS, and PVP capped silver nanoparticles at 404, 403, and 399 nm, respectively, which corresponds to the typical surface plasmon resonance (SPR) of spherical silver nanoparticles. When compared to citrate and SDS stabilized, the SPR peak of PVP stabilized nanoparticles was blue shifted towards a shorter wavelength of 399 nm. The shift in SPR is determined by the capping agent due to the local nature of its effect on the surface of the nanoparticles [28]. 

### 3.2. Transmission Electron Microscopy (TEM)

**Figure 2:**
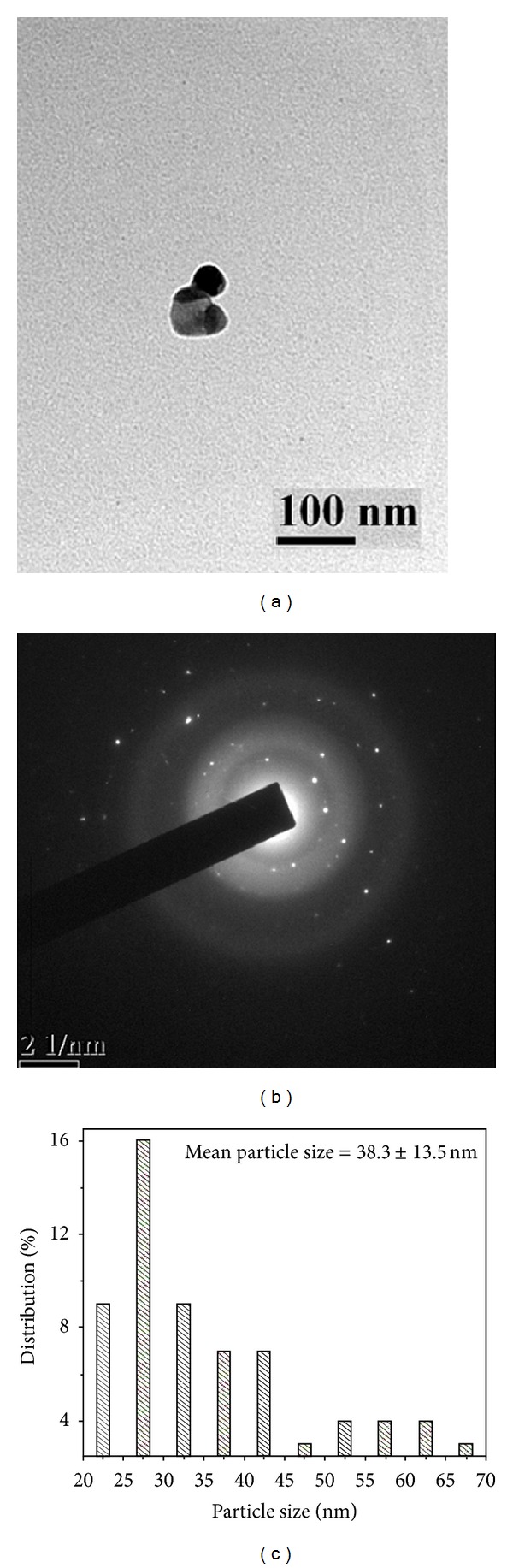
TEM images of citrate stabilized silver nanoparticle, at (a) 100 nm scale, (b) corresponding SAED pattern, and (c) histogram showing the particle size distribution.

**Figure 3:**
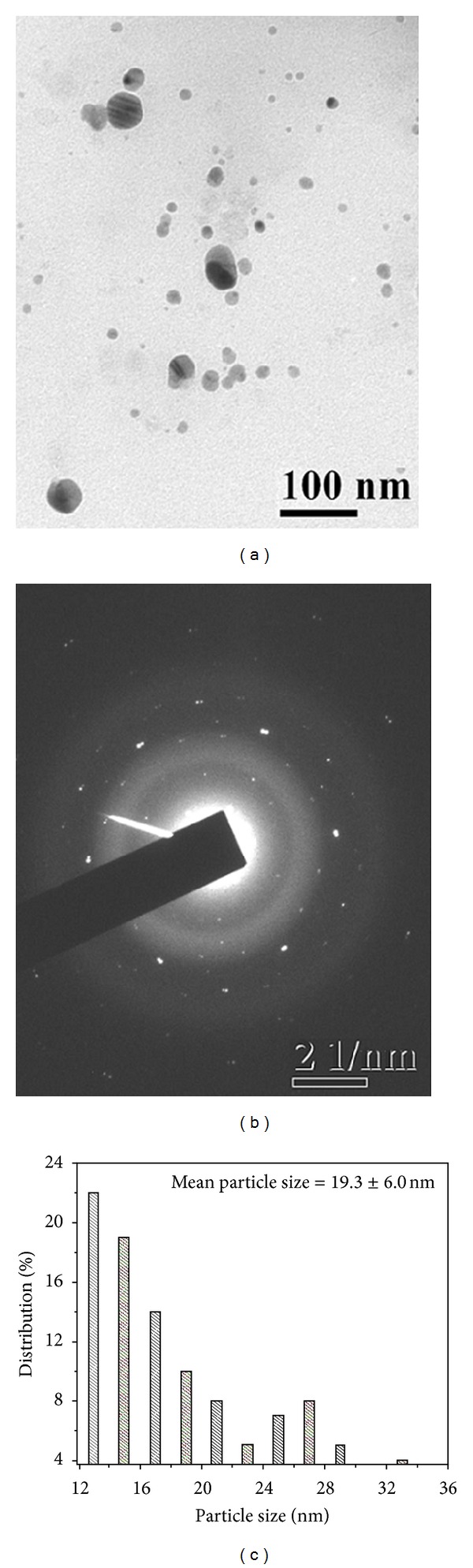
TEM images of SDS capped silver nanoparticle, at (a) 100 nm scale, (b) corresponding SAED pattern, and (c) histogram showing the particle size distribution.

**Figure 4:**
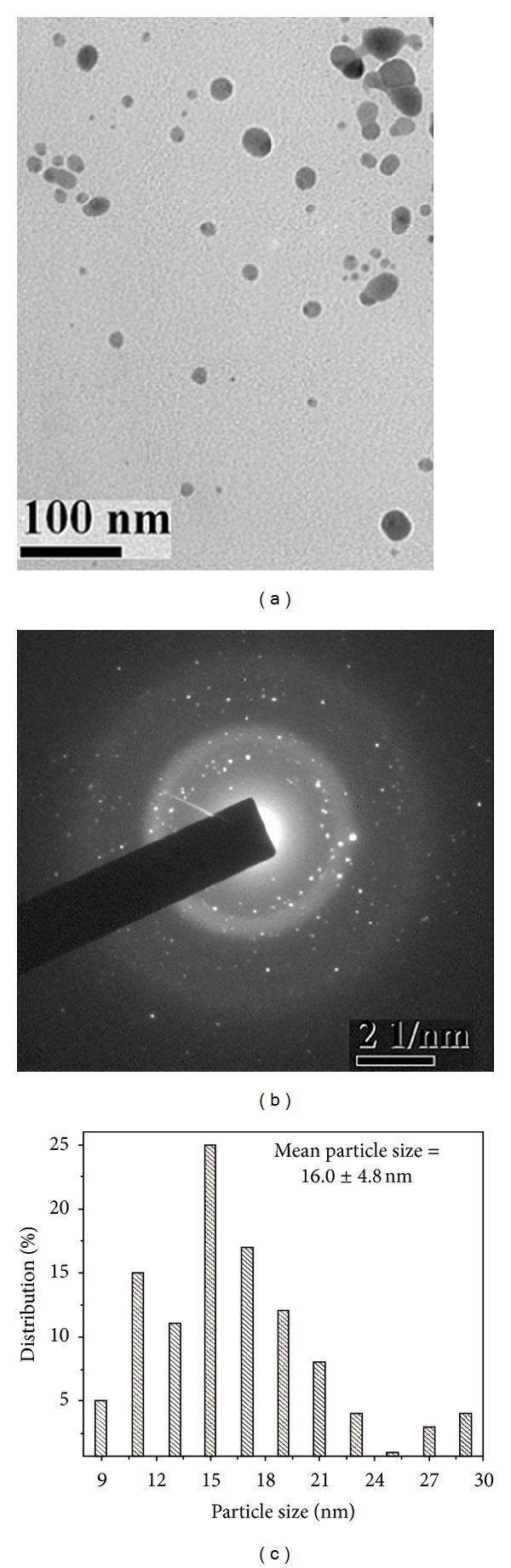
TEM images of PVP stabilized silver nanoparticle, at (a) 100 nm scale, (b) corresponding SAED pattern, and (c) histogram showing the particle size distribution.

Figure 2 shows the TEM image of the silver nanoparticles stabilized with citrate. These nanoparticles are mostly spherical and were few square shaped, polydisperse, and showing wide range of particles from 21 to 70 nm with bimodal distribution. The smaller sized population had a mean diameter of 31.1 ± 6.2 nm (73%), while the larger sized one had a mean diameter of 57.5 ± 7.8 nm (27%). The average particle size obtained from both populations was about 38.3 ± 13.5 nm (*P* ≤ 0.0001) (Figure 2(c)). Furthermore, the size of the particles was compared with SDS capped colloids (Figure 3). The nanoparticles are spherical in shape, polydisperse with bimodal distribution showing sizes of 12–34 nm. The smaller sized population had a mean diameter of 16.3 ± 3.1 nm (79%) and the larger sized one had a mean diameter of 28.1 ± 2.9 nm (21%). The average particle size of the two modes was about 19.3 ± 6.0 nm (*P* ≤ 0.0001) (Figure 3(c)). Furthermore, the size was also evaluated for PVP capped colloidal solution (Figure 4). The produced nanoparticles are spherical, nonaggregated, and bimodal with a size distribution of 8.0–28 nm. The smaller and larger sized populations had mean diameters of 15.1 ± 3.7 nm (92%) and 27.2 ± 1.1 nm (8%), respectively. The average particle size obtained from both the diameter distributions was about 16.0 ± 4.8 nm (*P* ≤ 0.0001) (Figure 4(c)). The bimodal distribution is likely due to the inhomogeneous growth, which is kinetically favoured. Among the preparations, the silver colloids stabilized with PVP exhibited a narrow particle size distribution. It is worth noting that with PVP, the average size of the nanoparticles formed decreased. The decrease in polydispersity and mean particle size with PVP stabilized particles was also evident from the TEM image. This is possibly due to the higher electron donating ability of PVP leading to a stronger interaction with positively charged silver ions during reduction, thereby an enhanced stabilization between capping molecules and nanoparticle surfaces [29]. The size and shape of the nanoparticles synthesized depend on many parameters such as choice of reduction technique, concentration of metal precursor, reductant, and capping agent [28].

### 3.3. X-Ray Diffraction (XRD)

**Figure 5:**
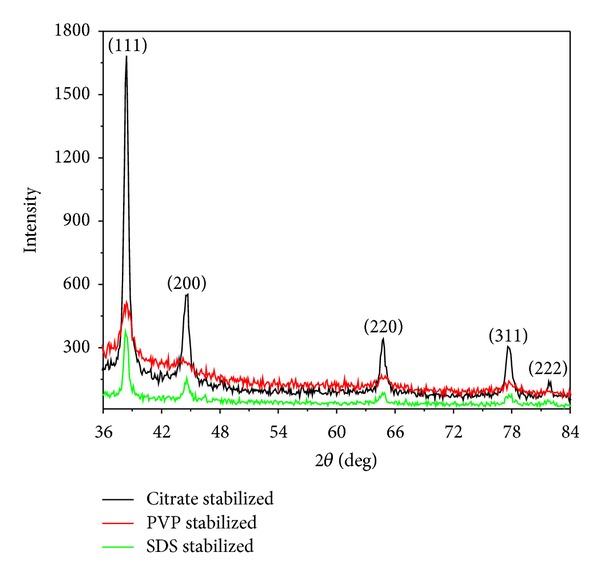
The XRD pattern of capped silver nanoparticles, indicating the face centered cubic (fcc) crystal structure.

The XRD pattern of the stabilized silver nanoparticles is shown in Figure 5. There were five well-defined characteristic diffraction peaks at 38.3°, 44.5°, 64.8°, 77.6°, and 81.8°, respectively, corresponding to (111), (200), (220), (311), and (222) planes of face centered cubic (fcc) crystal structure of metallic silver. The interplanar spacing (*d*_hkl_) values (2.307, 2.012, 1.437, 1.229, and 1.176 Å) and the lattice constant (4.047 Å) calculated from the XRD spectrum of silver nanoparticles are in agreement with the standard silver values (JCPDS PDF card 04-0783). Therefore, the XRD pattern further corroborates the highly crystalline nature of nanoparticles observed from the selected-area electron diffraction (SAED) patterns depicting concentric rings with intermittent bright dots (Figures 2, 3, and 4). In addition, the broadening of the diffraction peaks was observed owing to the effect of nanosized particles. From the diffraction pattern, it is clear that the lattice plane (111) is the favored orientation for the generated nanoparticles [30].

### 3.4. Antibacterial Activity

**Figure 6:**
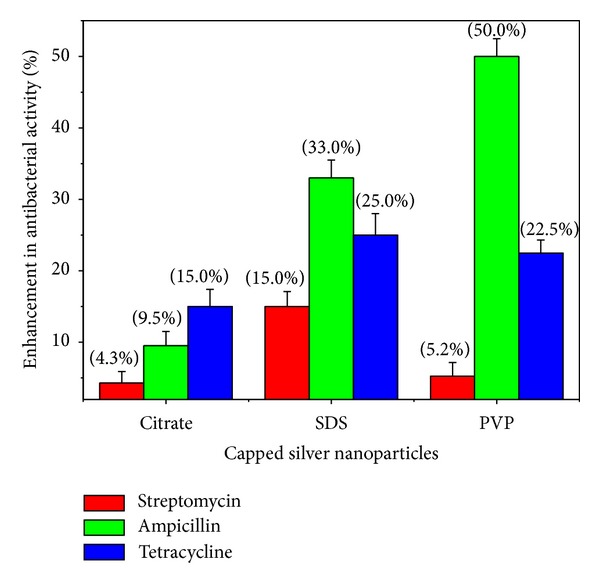
The percentage of enhancement in antibacterial activity observed for the antibiotics in combination with silver nanoparticles, against the bacterial strain *E. coli* ATCC 25922.

**Figure 7:**
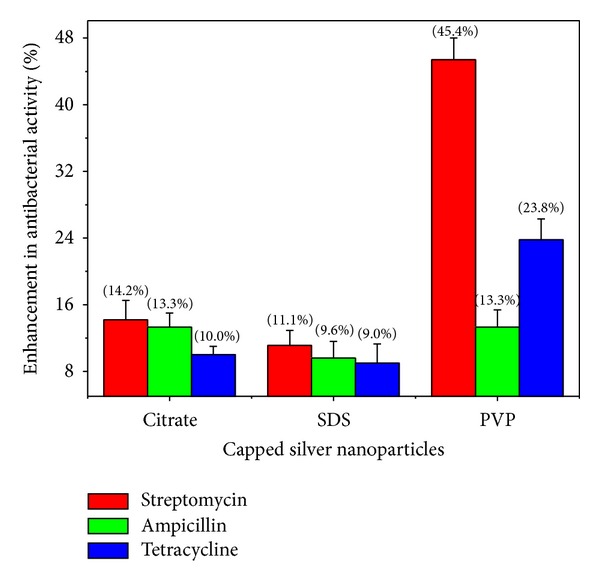
The percentage of enhancement in antibacterial activity observed for the antibiotics in combination with silver nanoparticles, against the bacterial strain *S. aureus* ATCC 25923.

In this report, the combined effect of the capped silver nanoparticles with antibiotics was assessed in comparison to antibiotics, with disc diffusion method. The concentrations of capping agents used for synthesis of nanoparticles did not show any antibacterial activity on the test strains. It was observed that the collective effect of silver nanoparticles with antibiotics was additive. The activity of the three antibiotics: streptomycin, ampicillin, and tetracycline with citrate, SDS, and PVP capped nanoparticles, against *E. coli* and *S. aureus* strains was shown as percentage enhancement in antibacterial effect. The increase in antibacterial activity of different antibiotics was quantified by the equation (*B* − *A*)/*A* × 100, where *A* and *B* are the ZOI for antibiotic and antibiotic + silver nanoparticles, respectively. The activity of all the tested antibiotics was increased in combination with all the silver nanoparticles employed, against the test bacterial strains. For the Gram-negative *E. coli*, the highest increase was noted for tetracycline (*P* ≤ 0.004) followed by ampicillin (*P* ≤ 0.01) and streptomycin (*P* ≤ 0.05) with citrate capped nanoparticles. For SDS capped nanoparticles, the raise in the activity was in the order of ampicillin (*P* ≤ 0.001)> tetracycline (*P* ≤ 0.001)> streptomycin (*P* ≤ 0.005). The highest percentage of enhancement was found for ampicillin (*P* ≤ 0.0004) followed by tetracycline (*P* ≤ 0.002) and streptomycin (*P* ≤ 0.03), with PVP capped nanoparticles (Figure 6). In the case of Gram-positive *S. aureus* strain, the order was found to be streptomycin (*P* ≤ 0.005)> ampicillin (*P* ≤ 0.01)> tetracycline (*P* ≤ 0.01) for citrate and SDS capped nanoparticles. For the PVP capped nanoparticles, the enhancement was in the order of streptomycin (*P* ≤ 0.0005)> tetracycline (*P* ≤ 0.001)> ampicillin (*P* ≤ 0.005) (Figure 7). Interestingly, among the selected antibiotics, streptomycin has shown the highest activity against *S. aureus* with PVP capped silver nanoparticles. The collective activity of nanoparticles capped with PVP with the antibiotics streptomycin and tetracycline against *S. aureus* was found to be the highest in comparison with citrate and SDS capped nanoparticles. With the same polymer capped nanoparticles against *E. coli*, the highest percentage of enhancement was observed for the antibiotic ampicillin (Figure 6). From the data, it is evident that antibiotic ampicillin demonstrates the highest percentage of enhancement in activity against *E. coli* with PVP and SDS capped nanoparticles. In contrast, the maximum increase in activity against *S. aureus* was observed for the streptomycin antibiotic with PVP capped nanoparticles. The differential susceptibility of Gram-negative and Gram-positive bacteria towards antibacterial agents possibly depends on their cell wall structure [31]. The results obtained in our study on collective effects of antibiotics with silver nanoparticles are in similar lines with earlier studies reported [21, 22, 24, 25, 32–34]. 

The data suggests that the enhancement in antibacterial activity of antibiotics with silver nanoparticles depends on the influence of the capping agent on nanoparticles. The combined effects of silver nanoparticles and antibiotics were more prominent with PVP capped nanoparticles as compared to citrate and SDS capped ones. This can be probably attributed to the steric stabilization of nanoparticles by the polymer PVP. It is well established that PVP capped silver and gold nanoparticles exhibit excellent stability towards changes in pH and ionic strength [35, 36]. Thus, the PVP capping on the surface of nanoparticles protects from changes in environmental conditions and prevents aggregation. In addition, the capping agent PVP is known to improve the bioavailability of the drug and the same is reported for curcumin conjugated to PVP capped gold nanoparticles [37]. The probable mechanism involved in enhanced antibacterial activity of antibiotics with silver nanoparticles can be attributed to the bonding reaction between nanoparticles and antibiotic molecules. The active functional groups of antibiotics such as hydroxyl and amino groups react with large surface area of the silver nanoparticles by chelation [22].

## 4. Conclusions

In this study, we have used a chemical reduction method for the synthesis of silver nanoparticles in the presence of three different capping agents. The produced nanoparticles were found to be spherical in shape and polydisperse. The antibacterial activity of the selected antibiotics was increased in the presence of these capped silver nanoparticles against test strains. The increase in activity was more pronounced with PVP capped silver nanoparticles for both Gram-negative and Gram-positive bacteria. In view of this, further studies are envisaged to explore the mechanism involved in enhanced antibacterial activity.
